# The Severer Forms of Laryngitis

**Published:** 1920-02-14

**Authors:** 


### THE SEVERER FORMS OF LARYNGITIS.

### I. Early Diagnosis and Treatment.

In dealing with so wide a subject, and one involving so many secondary considerations, it is difficult to compress the essential points into the confines of two short articles without the result being a mere tabulation of facts, but with a localisation which presents such natural difficulties in examination and which may in its early stages be of either the severest or most simple portent, one need offer no excuse for bringing reminders of some of the salient points to the practitioners' and the nurses' notice. It is the simple signs or rather their correlation which form the keynote to success in diagnosis, and with this fact in view, the most outstanding are set out below.

### 1. Acute Catarrhal Laryngitis.

*History*: Recent "cold" in the head; influenza, or other specific fever; exposure to infection, through occupation, etc.; previous attacks; excess of alcohol or tobacco.

*Symptoms*: Discomfort in the region of the larynx; tickling, irritating cough, which at this stage is dry, with very little expectoration. Very soon there will probably be a varying amount of mucus secreted, which is sometimes bloodstained. Marked huskiness and often complete aphonia; dysphagia, and even dyspnœa, in very severe cases, which sometimes occur in sudden attacks at night. This latter condition is specially marked in children when the disease is known as laryngitis stridulosa, which forms one variety of "croup."

*Examination*: In every case of laryngitis the condition of the nose, nasopharynx, and pharynx should first be ascertained; catarrhal affections of the larynx being so very often secondary to lesions of the higher portions of the respiratory tract.

Larynx shows marked congestion and redness of the cords, with some restriction in their movements. The mucus membrane over the aryteno-epiglottic folds is thickened and may form pyriform swellings, which, if very marked, can reduce the rima glottidis to a mere chink.

The crico-arytenoid articulations may be actually inflamed, and then there will be distinct impairment of the movements of the cords.

Pain on pressure over the larynx; pain and a grating sensation on moving the larynx *en masse* from side to side.

The diagnosis in the adult is easy, because a laryngoscopic examination can be made without very much difficulty, but in a child this is generally impossible; and in this latter class of case the medical man must eliminate the question of true laryngismus stridulosa (spasmodic croup). In acute catarrhal laryngitis the disease generally begins with coryza; fever, hoarseness, and cough are present; if dyspnœa be present it is generally more marked; there are no carpo-pedal contractions.

The diagnosis from acute membranous laryngitis is a very difficult matter unless membrane be actually coughed up, and this is not a common event; in this latter disease stenosis, and therefore dyspnœa, are much more pronounced, causing a much more extensive and powerful use of the muscles of forced respiration; again, cyanosis occurs more readily.

From primary laryngeal diphtheria the diagnosis is again a most difficult matter, but in the usual type of this disease one can generally see a membranous deposit on the pharyngeal, or perhaps the nasal mucous membranes.

In diphtheria there is early swelling of the lymphatic glands below the angles of the lower jaw, and albuminuria is not uncommon; also this disease is of a more asthenic type. However, in an early case it will be wise to withhold a definite diagnosis at the outset.

#### Treatment.

A. *Adults*: When unaccompanied by sepsis the condition is not generally a very severe one. Rest to phonation and respiration, viz., keep the patient in bed in a constant temperature of about 65° F., the air being kept moist by means of a bronchitis kettle containing tinct. benzoini co., ʒi. to 1 pint. Give inhalations of menthol (menthol grs. 5 to sp. vini rect. ʒi; one teaspoonful (viz., ʒi) to a pint of water, half boiling and half cold) for ten minutes every three hours, or even oftener.

An inhaler containing ammonium chloride may be used instead, if preferred. If the condition be not very severe, an oral and laryngeal spray of menthol 20 per cent. in paroleïn may be used with very great advantage; one of the best atomisers is the de Vilbiss variety, as the end-piece can be turned in the required direction with the minimum of trouble, and the nozzle, being made of metal, can easily be sterilised.

For the fever a diaphoretic mixture may be given, and troch. morph. et ipecac. or troch. krameria et cocaine will relieve the irritating cough. The treatment of intra-laryngeal œdema will be found under that occurring in acute septic laryngitis. It is never a wise plan to attempt to cut an attack short by the use of strong caustics; especially is silver nitrate a dangerous drug to use inside the larynx.

B. *Children*.—Treatment should be on the same general lines as indicated for adults, but there are one or two modifications to be recommended; thus it is a good plan to give a hot bath at the outset, and to administer a purge of castor oil or calomel; place a hot sponge over the neck, and feed with warm, diluted milk or albumen water.

If there should be any tendency to spasm, give small doses of potass. brom. with your diaphoretic mixture; if dyspnœa should increase, then administer ipecacuanha until vomiting occurs, or else inject apomorphine subcutaneously. If still there is no relief and cyanosis, etc., is coming on, then resort to intubation, or, if this be impossible, perform high tracheotomy.

A good prophylactive treatment against the occurrence of suffocative laryngeal spasm, or "croup," is to wake the child frequently during the night and persuade it to swallow a mouthful of some warm, demulcent drink; it will not disturb the little patient to any extent.

### 2. Acute Traumatic Laryngitis.

There will probably be a history of patient having inhaled steam, or children drinking from a spout of boiling water; of drinking corrosive poison; or a foreign body may have found its way into the larynx; or of caustics having been applied to the interior of the larynx, or its immediate neighbourhood.

*Diagnosis*: Symptoms and signs occur very rapidly, œdema being early on the scene.

Treatment must be proportionately more rapid and

**The Severer Forms of Laryngitis**—(*continued*).

radical than in (I.), or else a fatal result is not at all unlikely. Scarification of the œdema is especially indicated in these cases. Other treatment will be on similar lines to (I.), but, and especially in children, early tracheotomy may have to be performed; also ice packs, iced drinks, ice pellets to suck, are very useful. It is a good plan to give nothing else by the mouth for the first twenty-four hours. Scarification, if done early, may save the patient from tracheotomy.

3. Acute Epiglottiditis.

*Diagnosis.*—The condition may be limited to the epiglottis, or be part of a general laryngitis. A frequent cause is influenza (this complication seemed to be a fairly common one during the late severe epidemics), or the lesion may be of septic origin.

A swollen epiglottis gives rise to a typical voice; it is thick and full, but there is no huskiness unless the cords be also involved; the patient complains of a sensation of a "lump in the throat" when swallowing, and often also of an inclination to vomit; as a rule there is a profuse secretion of mucus.

On examination there is generally evidence of pharyngitis or laryngitis, or both, but in some cases the inflammation is entirely limited to the epiglottis and its immediate neighbourhood. The latter appears greatly congested, bright red or purple in colour, with a varying amount of swelling and often some sticky mucus. Fever is generally high, and there is marked malaise; it is very interesting to note what profound effects on the body, as a whole, can be produced by quite a small lesion. There may be tenderness over the hyoid bone, and the superior deep cervical and infra-hyoid, lymphatic glands may or may not be enlarged and tender.

*Treatment.*—The same as for (1), but in this case scarification is generally more called for; this is not a difficult, and is quite a painless, proceeding with the use of cocaine, a laryngeal lancet, and skill.

An aqueous solution of ichthyol $\frac{1}{2}$ per cent., used as a spray every quarter of an hour, has been strongly recommended by Mayjes for this and other closely allied conditions.

A spray of menthol 20 per cent. in parolein, on to the epiglottis, is excellent treatment; it relieves the congestion, is pleasant to smell, and therefore to taste, and leaves behind it a cold anæsthetic effect.

4. Acute Membranous Laryngitis.

*Diagnosis*: Children are by far the principal sufferers. It is most easily mistaken for primary laryngeal diphtheria; in both cases dyspnœa and cyanosis are early complications, but in diphtheria the cervical glands are enlarged at an earlier stage, and albuminuria is concomitant and more constant in its occurrence.

From a very severe case of acute catarrhal laryngitis the diagnosis is often most difficult, unless portions of membrane are coughed up, and this, as already stated, is not a common occurrence.

*Treatment*: On the same general lines as in (1); but intubation, or tracheotomy, is more likely to be necessary. Emetics are often indicated, whilst calomel, given internally, frequently, and in small doses, or as an inhalation by volatilising 15 grains, is a most valuable drug.

In every case where there is any doubt about the question of diphtheria being present it is much wiser to inject full doses of anti-diphtheritic serum at once, than to wait for events.

